# HIV Pre-Exposure Prophylaxis (PrEP) purchase patterns and STI occurrence among Israeli men: A cohort analysis

**DOI:** 10.1371/journal.pone.0259168

**Published:** 2021-11-18

**Authors:** Daniel Chemtob, Clara Weil, Jordan Hannink Attal, Elias Hawila, Enav Noff Sadeh

**Affiliations:** 1 Department of Tuberculosis and AIDS, Israel Ministry of Health, Jerusalem, Israel; 2 Braun School of Public Health and Community Medicine, Hebrew University of Jerusalem, Jerusalem, Israel; 3 Maccabi Healthcare Services, Tel Aviv, Israel; The Chinese University of Hong Kong, CHINA

## Abstract

**Background:**

HIV Pre-exposure prophylaxis (PrEP) is the regular use of antiretroviral medication by people who are not infected with HIV to prevent seroconversion. Israel approved PrEP for continuous use in 2017, and Israeli Health Maintenance Organizations (HMO) offered PrEP with a copayment to eligible members.

**Methodology:**

This retrospective cohort study included all people who were dispensed PrEP between September 2017 to June 2019 in the second largest HMO in Israel. Statistical analysis, including Kaplan Meier, was conducted to evaluate user PrEP purchase, adherence to medical follow-up, and clinical outcomes.

**Results:**

In total, a cohort of 757 PrEP users were followed for 657.8 person-years. All but one user were male; median age was 35 years. At baseline, 0.8% had gonorrhea and 1.5% had chlamydia infections and 4.4% had recent syphilis infection. Continuous use of PrEP (without interruption/discontinuation) was observed in 29.9%, while 39.9% interrupted and 30.3% discontinued use. Median time to first interruption/discontinuation was 4.0 months. At 6–12 months after initiation, 79.8% of users had a documented HIV test, 77.3% a Chlamydia-Gonorrhea panel, and 78.9% a creatinine test. There was one new case of HIV among the cohort, five months after PrEP discontinuation. Estimated first-year infection rates were 5.0%, 8.6% and 6.8% for gonorrhea, chlamydia and first-time syphilis, respectively.

**Conclusions:**

This study shows heterogeneous PrEP purchase patterns and required medical follow-up, and an increase in STIs among consistent PrEP users. Improving adherence to recommended medical follow-up during PrEP use is essential in PrEP’s integration into Israel’s national HIV prevention strategy.

## Background

Following proven efficacy in four randomized controlled trials [1–4], the World Health Organization (WHO) released guidance on HIV pre-exposure oral prophylaxis (PrEP) for serodiscordant couples, men and transgender women who have sex with men at high risk for human immunodeficiency virus (HIV) in 2012 [5]. Used regularly, PrEP can protect uninfected persons from acquiring HIV. In high-income countries, particularly those whose HIV endemics were concentrated among MSM, the effectiveness of PrEP in clinical trials posited a game-changer in HIV prevention strategies. However, in the years following WHO and Centers for Disease Control approval, PrEP uptake has been slow in many high-income countries [6, 7].

Two dosage regimens for PrEP were recommended. First, the continuous dosage regimen, which requires users to take pills daily. A second dosage regimen was developed, called ‘on demand’ that users take in anticipation of sexual encounters [8, 9].

Israel’s HIV incidence rate, 4.8 cases per 100,000 persons in 2018, is low, with a majority of cases concentrated among immigrants from endemic countries, men who have sex with men, and intravenous drug users [10, 11]. Between 2011–2015, MSM accounted for over 30% of all new HIV infections and 46% of new HIV cases among men notified to the Israeli Ministry of Health (MoH) [10]. Israel has universal healthcare for all citizens, who are registered in one of four Health Maintenance Organizations (HMOs).

Beginning in 2014, the Ministry of Health Department of Tuberculosis and AIDS (DTA) began the policy making process for PrEP in Israel. In June 2017, PrEP (as emtricitabine/tenofovir disoproxil fumarate) was included in the official drug registry in Israel using the continuous dosage regimen [12]. Clinical guidelines for PrEP prescription were developed alongside PrEP’s inclusion in Israel’s drug registry, and defined eligibility for PrEP as serodiscordant couples and high-risk MSM and trans people. Only physicians who took a seminar on PrEP prescription and management were authorized to prescribe PrEP. According to the guidelines, potential users seeking PrEP prescription are required to undergo preliminary tests, including for HIV, STIs, and creatinine before starting PrEP, and must adhere to medical follow-up [12–14].

As a result of PrEP entering the official registry, Israeli HMOs began to offer PrEP exclusively to their members who paid for voluntary supplementary insurance beyond the standard national insurance afforded to all citizens [13, 14]. As of 2011, 74% of Israeli adults have supplementary health insurance [15]. The copayment for a monthly supply of PrEP was set between 84–103 USD, dependent on the HMO and level of coverage [12].

This study presents a cohort of PrEP users who received PrEP through Maccabi Healthcare Services (MHS), the second largest HMO in Israel. The aims of this study were to characterize PrEP users, to assess PrEP purchase patterns, to assess adherence to medical follow-up, and to describe trends in STI incidence among PrEP users.

## Methods

This retrospective database cohort study was jointly planned and performed by the DTA and by MHS. MHS serves a quarter of the population in Israel. The MHS database contains longitudinal data on a stable population of more than 2.5 million people since 1992. Data are automatically collected and include comprehensive laboratory data from a single central lab, full pharmacy prescription and purchase data, and extensive demographic data on each patient.

The study population included all adults at least 18 years of age in MHS with at least one dispensed prescription of PrEP from September 1^st^ 2017 through June 31, 2019 (index period). Patients were identified among all designated health care clinics of MHS. Data were extracted using specific codes for the PrEP indication in the database. Data collection was conducted through August 2019.

### Study variables

Baseline data collected included patients’ sex assigned at birth and age as well as laboratory blood test results for serum creatinine and eGFR, viral infections (HIV, HBV and HCV) and sexually transmitted infections (gonorrhea, chlamydia, syphilis) were obtained up to a year before index date. Results were recorded according to the most recent valid test result before or on the index date. Results were reported as negative or positive or relative to standard normal ranges. In order to better understand the time of the most recent test, the proportion of patients tested within 90 days before index were also described.

STI incidence was defined by detection in urine using nucleic amplification tests for *Neisseria gonorrhea* and *Chlamydia trachomatis*. Syphilis serology was determined through chemiluminescent microparticle immunoassay, confirmed by rapid plasma reagin testing (‘Syphilis Ab’); positive samples were also tested with *T pallidum* hemagglutination assay (TPHA) and Venereal Disease Research Laboratory (VDRL). To investigate incidence of first-time syphilis infection, analyses focused on seroconversion among people who were negative for any syphilis antibodies at baseline. Incidence of syphilis among patients with a history of syphilis past infection at baseline was not captured in this study due to the complexity of analyzing titer changes and treatment patterns in a retrospective database study.

### Data analysis

Data were analyzed using two purchasing pattern classifications. In order to investigate if purchases were made often enough to facilitate a continuous regimen, PrEP purchase pattern categories was classified as follows:

- Interrupted usage: ≥60 days between purchases of PrEP (i.e. resumed treatment after a break)
- Discontinued usage: ≥60 days after the last purchase of PrEP in the study period (i.e. did not resume PrEP by the end of follow-up)
- No discontinuation or interruption in usage: ≤60 days between purchase of PrEP

To investigate the relationship between PrEP use and STI incidence, PrEP purchase patterns were also classified as:

- Discontinued after 1 purchase
- Discontinued or interrupted after >1 purchase
- No discontinuation or interruption in purchases

Using these purchase pattern categories allowed analysis of STI trends among men who used PrEP compared to those who discontinued PrEP after only one purchase.

In addition, adherence to medical follow-up was defined as performing the recommended laboratory tests within set time periods (12–26 weeks and 27–52 weeks) between the first dispensed PrEP and up to 30 days following the last PrEP supply within the study period. These time periods reflect clinical guidelines for PrEP use in Israel, which recommends medical follow-up every six months.

Descriptive statistics were presented as median (interquartile range [IQR]) for continuous variables and as frequencies for categorical variables. For laboratory tests with missing data, the distribution of results was described among the total population as well as among those tested (percentage among valid test results). Kaplan-Meier and Cox regression were used to assess time to HIV or STI infection acquisition. Statistical analysis was performed using SPSS (v.25). A p-value of <0.05 was considered statistically significant.

This study was conducted per approval of the Maccabi Research Committee and the institutional review board (IRB) of Bait Balev Hospital, and was performed in accordance with all relevant guidelines. Informed consent for this study was waived by the Maccabi Research Committee and IRB of Bait Balev Hospital on the grounds that the study was based on data in an existing database.

## Results

In total, 813 MHS members purchased at least one month’s worth of PrEP during the index period, of whom 1 was less than 18 years old and 56 were excluded due to enrolling in MHS less than 12 months before first purchase. In total, 757 MHS PrEP users were eligible for inclusion in the study, totaling 658.7 person years of follow-up.

In the study sample, 756 PrEP users were male (99.9%), and the median age was 35 years (IQR: 29–41). A majority of PrEP users were from the Tel Aviv-Yafo Metropolitan Area (493; 65.1%), followed by Jerusalem (n = 83; 11.0%).

### Baseline characteristics

At baseline, 97.2% of the sample population had a valid creatinine test in the previous year, and 95.7% had a result within normal range. A total of 729 (96.3%) users had a record of a HIV test performed in MHS in the last year at time of PrEP initiation, and between 91.3–93.9% had valid tests for STIs. Active STI at baseline stood at 11 (1.5%) positive for chlamydia, 6 (0.8%) positive for gonorrhea, and 33 (4.4%) positive for recent syphilis infection. In addition, 7.1% had evidence of past syphilis infection. Table 1 shows baseline test rates and results for all required tests before initiating PrEP.

**Table 1:** Baseline laboratory tests for cohort of PrEP users enrolled in MHS, Israel, 2017–2019*.

			N	% of Total	% of tested
HIV	Test rates	valid test in past year	729	96.3	
		valid test in past 90 days	701	92.6	
	Test results	Negative	729	96.3	100.0
		Positive	0	0	0
Creatinine	Test rates	valid test in past year	736	97.2	
		valid test in past 90 days	678	89.6	
	Test results	within normal range	704	93	95.8
		outside normal range	32	4.2	4.2
HCV Ab**	Test rates	valid test in past year	675	89.2	
		valid test in past 90 days	624	82.4	
	Test results	Negative	673	88.9	99.6
		Positive	1	0.1	0.1
		Borderline	2	0.2	0.3
HBV***	Test rates	valid test in past year	627	82.8	
		valid test in past 90 days	549	72.5	
	Test results	Negative	627	83.8	100.0
		Positive	0	0	0
Chlamydia	Test rates	valid test in past year	711	93.9	
		valid test in past 90 days	667	88.2	
	Test results	Negative	700	92.5	98.5
		Positive	11	1.5	1.5
Gonorrhea	Test rates	valid test in past year	711	93.9	
		valid test in past 90 days	667	88.1	
	Test results	Negative	705	93.1	99.2
		Positive	6	0.8	0.8
Syphilis	Test rates	valid test in past year	691	91.3	
		valid test in past 90 days	649	85.7	
	Test results	Negative	603	79.7	87.3
		Previous infection	54	7.1	7.8
		Active infection at time of test	33	4.4	4.8

*Baseline test results were based on the most recent test performed in MHS up to one year before PrEP initiation

**HCV Ab: Hepatitis C Antibodies

***HBV: Hepatitis B virus

### PrEP purchase patterns

By the end of the follow-up period, 226 people (29.9%) continued to consistently purchase PrEP without prior interruptions in use.

A total of 346 people (45.7%) discontinued PrEP by the end of follow-up. Median time until discontinuation was 16.6 months (95% CI: 14.8–19.2 months).

**Fig 1:**
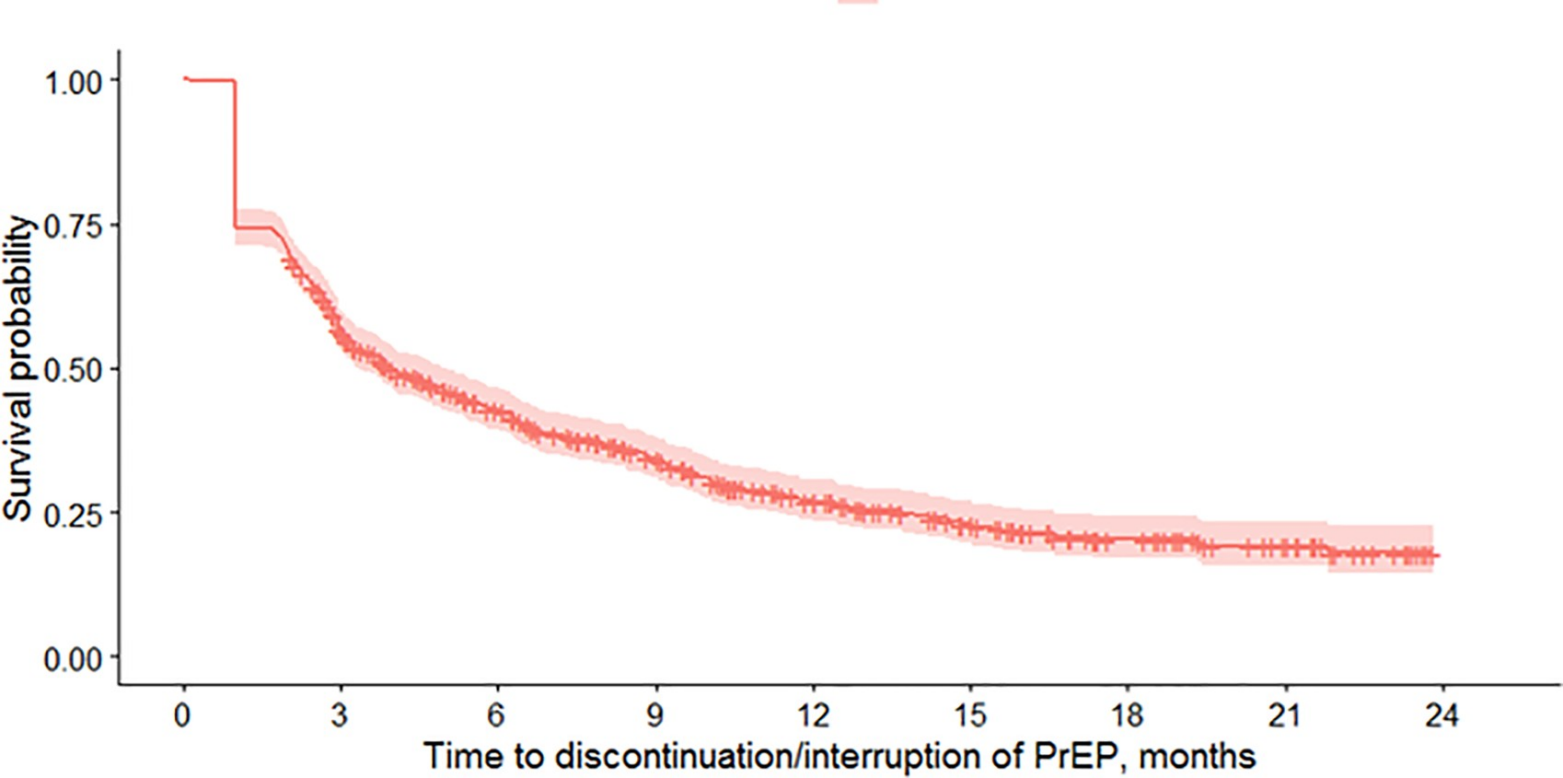
Interruption or discontinuation of PrEP (gap between purchases or after last purchase) through end of follow-up, (MHS, Israel, September 2017- August 2019).

In addition, 185 people (24.4%) interrupted and resumed PrEP usage before the end of follow-up. In total, 531 people (70.0%) interrupted or discontinued usage at any time during follow-up, with a median of 4.0 months (95% CI: 3.3–5.0 months) until the first interruption or discontinuation. The cumulative rates of interruption or discontinuation at 6 and 12 months were 57.2% (95% CI: 54.4–60.7) and 73.2% (95% CI: 29.4–76.5%), respectively (Fig 1). Discontinuation of PrEP after only 1 purchase was observed in 109 people (14.4%), while 226 (55.7%) interrupted or discontinued after 2 or more purchases.

### Adherence to medical follow-up

Overall, medical follow-up six months to one year after initiation for users who did not interrupt or discontinue PrEP varied by test, ranging between 52.5% for HBV to 79.8% for HIV. Adherence to recommended medical follow-up while using PrEP differed by patterns of use of PrEP; users who did not use PrEP continuously were less likely to perform follow-up testing. Table 2 presents the percentage of users who had follow-up medical tests during the period of PrEP use, by purchase pattern group.

**Table 2:** Medical follow-up among PrEP users (MHS, Israel, September 2017- August 2019)*.

		Chlamydia/Gonorrhea	Syphilis	HIV	HBV	HCV	Creatinine
		%	%	%	%	%	%
No interruptions or discontinuations	13–26 weeks after initiation (n = 210)	63.8	62.4	67.1	37.6	55.7	65.7
	27–52 weeks after initiation (n = 155)	84.5	83.9	85.2	57.4	76.1	83.9
No interruptions, then discontinued	13–26 weeks after initiation (n = 124)	37.9	39.5	46.8	29.8	37.9	46.0
	27–52 weeks after initiation (n = 48)	70.8	66.7	72.9	43.8	60.4	68.8
Interrupted, then resumed treatment	13–26 weeks after initiation (n = 185)	63.8	62.7	70.3	45.4	58.4	65.9
	27–52 weeks after initiation (n = 174)	80.5	79.3	84.5	56.3	72.4	84.5
Interrupted, then discontinued	13–26 weeks after initiation (n = 117)	59.0	54.7	62.4	39.3	48.7	61.5
	27–52 weeks after initiation (n = 107)	64.5	62.6	67.3	43.0	56.1	67.3

*The follow-up period was defined as the period between first dispensed PrEP prescription until 30 days after runout of the last PrEP dispensation in follow-up, through August 2019. The denominator was defined as the number of PrEP users actively enrolled at the start of a given time window.

When analyzing the number of STI tests during the period of PrEP use by person years, there were a total of 3.1 chlamydia/gonorrhea tests performed per person year of follow-up, and 2.8 syphilis tests performed per person year of follow-up. The number of STI tests per person year of follow-up differed by purchase pattern, with those without interruptions having the highest number of tests per person year of follow-up. Table 3 presents tests per person year by all three defined purchase patterns.

**Table 3:** STI tests per person year by purchase pattern (MHS, Israel, September 2017- August 2019).

		Purchase Patterns			Total
		Discontinued after 1 purchase	Interrupted or discontinued after >1 purchase	No interruption	
Total Users		109	422	226	757
Total Person Years on PrEP*		19.1	436.3	203.4	658.7
N tests	Chlamydia DNA/ Gonorrhea PCR	28	1247	783	2058
	Syphilis panel	23	1155	697	1875
Tests per person year	Chlamydia DNA/ Gonorrhea PCR	1.5	2.9	3.8	3.1
	Syphilis panel	1.2	2.6	3.4	2.8

*The follow-up period was defined as the period between first dispensed PrEP prescription until 30 days after runout of the last PrEP dispensation in follow-up, through August 2019.

### Sexually transmitted infections

**Fig 2:**
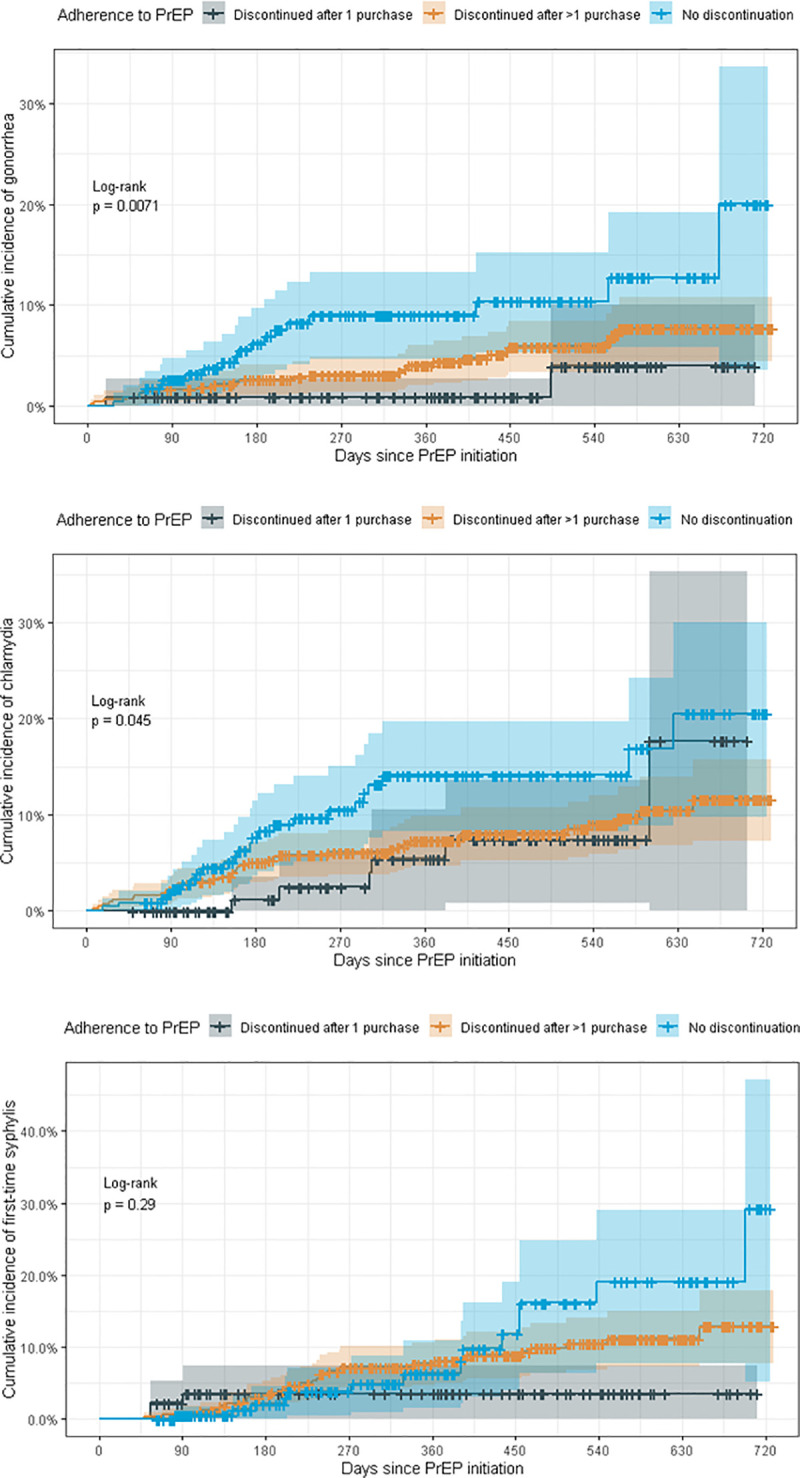
Time from PrEP initiation to STI through August 2019. **a:** Time from PrEP initiation to STI through August 2019 –Gonorrhea. **b:** Time from PrEP initiation to STI through August 2019 –Chlamydia. **c:** Time from PrEP initiation to STI through August 2019—First-Time Syphilis.

In this study, HIV seroconversion was not reported for any PrEP users while using PrEP. However, one PrEP user was diagnosed with HIV five months after discontinuing PrEP. Additionally, there were no new cases of Hepatitis B or C infection during follow-up. After one year of follow-up, the cumulative incidence of chlamydia excluding baseline values among the cohort was estimated to be 8.6% (95% CI: 6.4%-10.8%), the cumulative incidence of gonorrhea was 5.0% (95% CI: 3.3%-6.6%), and the cumulative incidence of first time syphilis infection syphilis was 6.8% (95% CI: 4.5%-9.0%). Fig 2A–2C show time to STI infection for gonorrhea, chlamydia, and first time syphilis, by PrEP purchase patterns. For gonorrhea and chlamydia, there was a significant difference in the rate of infection by the end of follow-up among those who continuously used PrEP compared to those who discontinued.

## Discussion

### PrEP purchase patterns

In order for PrEP to be an effective HIV prophylactic, users must adhere to a proper regimen [1, 13, 14]. This study observed purchase patterns to determine if users were purchasing PrEP often enough to facilitate adherence to a continuous regimen. A systematic review of adherence to PrEP has concluded that adherence varies drastically across settings and populations, with low adherence associated with start-up symptoms, low-risk perception, dosage regimen, and policy factors [16]. In the current study, PrEP users had heterogeneous patterns of use, with some patients adhering to the continuous PrEP regimen while others interrupted and/or discontinued use after a median of 4 months. In Israel, two possible contributing factors to this pattern of adherence are hypothesized- the high copayment as a potential barrier to long-term usage and the preference for an on-demand regimen.

During the study period, PrEP was only available at subsidized costs for people who paid for voluntary supplementary insurance via their HMO [17]. For these users, PrEP required a copayment of 84–103 USD [12] per one-month supply of a continuous regimen. Considering most medications covered by HMOs require minimal co-payments, the co-payment for PrEP is markedly high. Out-of-pocket costs could present a barrier both to people who initiated and can’t afford to continue taking PrEP and a deterrent for those who believe they would benefit from PrEP but cannot afford a high copayment. At the time of study, it was assumed that full integration into Israel’s national health insurance package (“basket of care”) would mitigate financial concerns of users and potential users to some degree.

However, in January 2020, PrEP was integrated into Israel’s “basket of care” and made available for all HMO members, rather than only those with voluntary supplementary coverage. As Israel has universal healthcare for citizens, this increased PrEP’s availability to all citizens through their basic health insurance [18]. Despite the State obtaining PrEP at a negotiated lower price similar to that in other European countries [16], the HMOs continue to charge a copayment of 84–103 USD (personal communication, Chen Shmilo, Israel AIDS Task Force, April 2020). Despite its integration into the basket of care, PrEP remains expensive for users themselves, and may continue to deter people from starting or continuing PrEP use.

Another potential explanation for frequent interruptions and discontinuations may be that users on a continuous prescription are using PrEP on an on-demand dosage regimen. The on-demand regimen was shown to be equally effective as a continuous regimen with high levels of adherence [19]. In addition, some studies have shown that the reduced pill burden of an on-demand regimen is associated with better overall adherence when compared to the continuous regimen [2]. Offering both regimens for users to decide which best matches their lifestyle is recommended to increase overall adherence [2]. While the current guidelines only include a continuous regimen, the MoH-DTA supports the addition of an on-demand regimen, and plans to update the national guidelines to reflect approval for both regimen dosages.

Obstacles to obtaining PrEP or avoidance of mandated medical follow-up may lead some users to outsource their PrEP prescriptions. In a study of PrEP users in Germany, 17.4% of PrEP users obtained PrEP through informal means, often from sources abroad [20]. The same study found that informal PrEP users were more than three times more likely not to adhere to medical follow-up [20]. Likewise, pathways to informally obtaining PrEP exist in Israel, though the extent of which is unknown. In order to conduct proper medical follow-up among PrEP users, Israel’s PrEP availability, accessibility, and regimen type must meet potential users’ abilities and needs.

Additionally, while physicians prescribing PrEP received training in prescription protocols, the extent to which physicians can perform adherence counselling is unknown. In other settings, different interventions have been used to increase adherence, including reminder calls or messages via telephone, printed materials, increasing the number of pill packs prescribed at one time, and trained counselors and nurses conducting individual sessions with users [21, 22]. Incorporating PrEP adherence counseling in the national HIV prevention strategy may improve adherence in the Israeli context, strengthening the utility of PrEP use in HIV prevention.

### Adherence to medical follow-up and STIs

In the present study, the incidence of gonorrhea and chlamydia during the study period varied by patterns of PrEP use, with a higher incidence observed among those who used PrEP continuously compared to those who discontinued after 1 months’ supply. Some studies have indicated that the incidence of STIs may increase during PrEP use due to increased testing during mandated follow-up while using PrEP [23]. However, the present study showed that adherence to recommended STI testing every six months was inconsistent, indicating the rise may be partially attributed to sexual behavior changes and not solely increased testing. Despite this finding, the incidence of gonorrhea and/or chlamydia are low compared to MSM taking PrEP in other high income countries [2, 20, 21]. Though Israel has lower incidence of gonorrhea, chlamydia, and syphilis than other high income countries, the number of cases among MSM are notably higher than the general population [24, 25]. The baseline and incident cases of syphilis within this limited study population are high compared to the absolute number of syphilis cases among MSM in Israel in previous years [24, 25], indicating the potential for syphilis outbreaks among PrEP users who do not use condoms to prevent the transmission of other STIs.

STI testing in the PrEP follow-up care framework is test to treat. In a meta-analysis of STI rates among MSM PrEP users, there was no substantial evidence that STI testing reduced the prevalence of chlamydia and gonorrhea [26]. For individual PrEP users, this implies that regular medical follow-up is paramount for individual health.

While this study shows a relatively higher incidence of non-HIV STIs among continuous PrEP users compared to those who discontinued after 1 months’ supply, the authors caution using ‘risk compensation’ to characterize this phenomenon. ‘Risk compensation’ for PrEP is the notion that increased PrEP uptake will lead to increased non-HIV STIs among PrEP users. The mechanism of this increase, according to risk compensation, is that PrEP users do not perceive increased risk (i.e. possibility to contract non-HIV STI), and therefore do not modify potentially harmful behavior (i.e. not using condoms). While risk compensation has been used to explain the rise in STIs in some PrEP studies, it has also been criticized as an unfitting theoretical model for sexual health because it relies on a rational model of human behavior found in economics rather than more complex models found in health promotion and bio-behavioral change theories [27].

The emergence of an STI does not negate PrEP’s effectiveness in preventing HIV infection, and PrEP provides users a medical framework to increase STI testing. The potential for increased incidence of STIs among PrEP users is a manageable phenomenon, and the current PrEP prescription and medical follow-up guidelines are appropriate for detecting STIs among users. The present study’s finding that adherence to STI testing in medical follow-up decreased over time is therefore concerning, and indicates the need for outreach to ensure that physicians are requiring testing and users understand the importance of regular STI panels.

### Strengths and limitations

This is the first study, to our knowledge, which analyzes a full cohort of PrEP users in Israel. The present study was conducted from a complete cohort data set in second largest HMO. The completeness of data, including all demographic and clinical data as well as purchase history, contributes to the reliability of the study. Since all PrEP users during the study period were included, results are generalizable for Israeli men who can afford the copayment and have geographical and temporal access to doctors accredited to prescribe PrEP.

There are a number of limitations to the present study. First, this study observed purchase patterns to determine if users were purchasing PrEP often enough to facilitate adherence to a continuous regimen. However, purchase does not equate to usage, and therefore does not necessarily accurately reflect adherence. Second, even considering the completeness of data in the study, many factors that contribute to PrEP purchase patterns and adherence to medical follow-up are entangled at the intersection of wide ranging personal, communal, social, and economic discourses that were not measured in the study. As such, this study can only offer theoretical explanations for PrEP usage patterns and follow-up. Furthermore, this study used purely descriptive statistics, which limits interpretation of data presented. In future studies, more rigorous statistical testing should be used.

Third, while all Israeli citizens have access to the same ‘basket of care’ regardless of which HMO they choose, service availability may differ between HMOs. In MHS, many PrEP prescribing physicians work in independent practices without additional support staff. As such, PrEP users in MHS may have been expected to schedule an additional appointment for blood tests, or received less explanations about PrEP from their doctor or clinic staff than PrEP users in other HMOs.

Lastly, this study shows significant differences in rates of chlamydia and gonorrhea infections for PrEP users who continuously used PrEP compared to those who discontinued, but not in first-time syphilis infections. This study was limited in its ability to capture recurrent syphilis and investigate syphilis treatment patterns. A second study using data collected in the present study, including medical chart review, will be conducted to assess the number of active recurrent syphilis cases among users during follow-up.

## Conclusion

PrEP is only effective as part of national and global HIV prevention strategies if users adhere to the prescribed regimen. This study shows that patterns of continuous regimen PrEP usage in Israel are heterogeneous. Medical follow-up likewise varied by user purchase and laboratory test. These results indicate the need to further explore the mechanisms for PrEP uptake and usage in Israel.

Users prescribed a continuous regimen that subsequently adopted an on-demand regimen may partially explain purchase patterns observed in this study. Analyzing purchase and adherence to an on-demand regimen first requires inclusion in the MoH clinical guidelines for PrEP prescription and usage. Considering subpar adherence to medical follow-up, including among those who used PrEP continuously without interruptions or discontinuation, doctors prescribing PrEP may require a refresher course, and supportive mechanisms integrated into electronic medical records may improve adherence to follow-up testing. From a broader perspective, high copayments for PrEP are currently addressed on a government level, and adherence counseling for PrEP users may improve adherence to PrEP and medical follow-up.

## List of abbreviations

DTA: Department of TB and AIDS
HIV: Human Immunodeficiency Virus
HMO: Health Maintenance Organization
MHS: Maccabi Health Services
MoH: Israel Ministry of Health
MSM: Men who have sex with men
PrEP: Pre-exposure prophylaxis
STI: Sexually Transmitted Infection
WHO: World Health Organization
